# Effect of an Herbal Mouthwash Derived from Pedicough Syrup on Plaque, Gingival and Bleeding Indices in Patients with Gingivitis: A Randomized Controlled Trial 

**DOI:** 10.30476/dentjods.2025.106004.2622

**Published:** 2026-06-01

**Authors:** Farin Kiany, Mohammad Ali Farboodniay Jahromi, Maedeh Ghorbani, Soroush Talakesh, Reyhaneh Ebrahimi

**Affiliations:** 1 Oral and Dental Diseases Research Center, Dept. of Periodontics, School of Dentistry, Shiraz University of Medical Sciences, Shiraz, Iran.; 2 Medicinal Plants Processing Research Center, Shiraz University of Medical Sciences, Shiraz, Iran.; 3 Dentist, Shiraz, Iran.; 4 Dept. of Periodontics, Dental Implants Research Center, Dental Research Institute, School of Dentistry, Isfahan University of Medical Sciences, Isfahan, Iran.; 5 Dept. of Periodontics, School of Dentistry, Shiraz University of Medical Sciences, Shiraz, Iran.

**Keywords:** *Thymus Vulgaris*, *Hedera Helix*, *Althaea officinalis*, Chlorhexidine, Mouthwash

## Abstract

**Background:**

Gingivitis is a reversible periodontal disease caused by plaque. It can be prevented and managed through mechanical and chemical plaque control methods. While chlorhexidine (CHX) is the gold standard mouthwash, its side effects have led to interest in herbal alternatives. Herbal mouthwashes containing plants such as *Thymus vulgaris, Hedera helix*, and *Althaea officinalis* show promise for treating gingivitis.

**Purpose:**

This study evaluates a novel herbal mouthwash, derived from Pedicough syrup, as an adjunct to scaling and root planning (SRP) for chronic gingivitis treatment.

**Materials and Method:**

This double-blind randomized clinical trial included 45 systemically healthy individuals diagnosed with generalized chronic gingivitis. All the participants received phase 1 of periodontal treatment (oral hygiene instruction and SRP). They were then randomly assigned to three groups regarding the mouthwash they used as Group 1 (CHX mouthwash), Group 2 (Pedicough mouthwash), and Group 3 (placebo). Participants used 15ml of their assigned mouthwash twice daily for two weeks. At baseline and two weeks after using the mouthwashes, a single blinded operator assessed clinical periodontal parameters including gingival index, bleeding index, and plaque indices, which were evaluated across the entire mouth. Data analysis was conducted using SPSS software with statistical significance set at *p* Value < 0.05.

**Results:**

All groups demonstrated improvements in indices from baseline to the end of the study. The two test groups showed greater reductions in plaque, bleeding, and gingival indices compared to the placebo group, and these differences were statistically significant. However, no significant differences were observed between the test groups in terms of periodontal parameters.

**Conclusion:**

The new herbal mouthwash demonstrated beneficial effects on clinical periodontal parameters, including plaque, gingival, and bleeding indices, comparable to those of CHX when used as an adjunct to SRP in patients with gingivitis.

## Introduction

Gingivitis is a reversible, non-destructive form of periodontal disease; the plaque-induced form is the most common [ 1 - 2 ]. It is caused by microbial plaque accumulating near the gingival sulcus. Local and systemic factors can increase plaque formation or make gingival tissue more susceptible to inflammation and infection [ 3 ]. In order to prevent or control plaque formation, it is essential to primarily follow mechanical methods like brushing and flossing [ 4 ]. Additional approaches, including the consumption of antiseptics [ 5 ], and herbal extracts [ 6 ], are often necessary to enhance oral hygiene.

Mouthwashes serve as topical agents to manage oral conditions like halitosis, gingivitis, and periodontal disease [ 7 ]. They complete mechanical plaque control, which requires skill and can be time-consuming [ 8 ]. Chemical mouthwashes, such as those approved by the American Dental Association (ADA), like chlorhexidine (CHX) and essential oil-based rinses, effectively target oral biofilm [ 9 ]. While CHX remains the gold standard and offers superior antibacterial effects, its side effects, including tooth discoloration and taste disturbances, have prompted interest in alternatives [ 10 ]. Essential oil mouthwashes are gentler but less effective at controlling plaque [ 11 ]. 

Herbal mouthwashes provide a natural alternative, utilizing plants with antibacterial, anti-inflammatory, and analgesic properties [ 12 ]. Notable examples include *Salvadora persica* (Miswak) [ 13 - 14 ], *Thymus Vulgaris* (*T. vulgaris*) [ 15 ], *Hedera Helix* (*H. helix*) [ 16 ], Punica granatum [ 17 ], and *Althaea officinalis* (*A. officinalis*) [ 18 - 19 ], which have shown promise in reducing gingivitis and tooth loss. Current evidence supports herbal mouthwashes as a promising adjunct in periodontal therapy, though further research is needed to confirm their plaque-reducing efficacy [ 20 - 21 ].

Some Studies have highlighted the potential of herbal products. For instance, Skrinja *et al*. [ 22 ] demonstrated that a mouth spray derived from *Althaea* reduces dry mouth symptoms and improves quality of life. Mojtahedzadeh *et al*. [ 23 ] found that Matrica and Persica herbal mouthwashes were as effective as CHX in improving periodontal indices. Research by Mahboubi *et al*. [ 18 ] showed that *A. officinalis* exhibits antibacterial properties against *Porphyromonas gingivalis, Fusobacterium nucleatum,* and *Actinomyces odontolyticus*. Bonaterra *et al*. [ 19 ] further confirmed the anti-inflammatory and antioxidative properties of *A. officinalis* in an herbal pill (Phytohustil), reinforcing its therapeutic potential.

Fani *et al*. [ 24 ] highlighted the strong antimicrobial potential of *T. vulgaris* oil against key oral pathogens, suggesting its use in oral care products or aromatherapy for preventing and treating oral infections. Süleyman *et al*. [ 25 ] demonstrated that H.helix exhibits a strong anti-inflammatory effect on both acute and chronic inflammation models in rats.

This study aimed to assess the effects of an herbal mouthwash formulated from three plants (*T. vulgaris*, *H. helix* and *A. officinalis*) as a supplementary treatment to scaling and root planing (SRP) for managing chronic gingivitis. The innovative mouthwash was developed using Pedicough syrup as its base. 

## Materials and Method

This study was a randomized, double-blind, controlled clinical trial conducted at the Periodontology Department of Shiraz Dental School. It included 45 systemically healthy participants diagnosed with generalized chronic gingivitis and employed a parallel group design. The study received ethical approval from the Ethics Committee of Shiraz University of Medical Sciences (Approval Code: IR.SUMS.REC.23472). It was registered on the Iranian Registry of Clinical Trials website (Registration Number: IRCT20211130053233N1). The trial was conducted in accordance with the consolidated standards of reporting trials (CONSORT) guidelines. Systemically healthy adults with generalized chronic gingivitis were recruited. Exclusion criteria were defined as smoking, pregnancy, medical conditions interfering with periodontal treatment, SRP within 3 months, use of antibiotics in the past month, or attachment loss (indicating periodontitis).

### Fabrication of Mouthwashes

### Pedicough Mouthwash

100mL of Pedicough syrup (Dineh, Iran; containing *T. vulgaris* extract 1.5mL, *H. helix* leaf extract 125mg, and *A. officinalis* root/flower extract 62.5mg per 5mL) was diluted with 55mL distilled water (total 155mL). The resulting 55% solution contained per mL: *T. vulgaris* extract 0.19mL, *H. helix* 16.13mg, and *A. officinalis* 8.06mg. 

### Placebo Mouthwash

155mL contained carboxymethyl cellulose (inert thickener; 0.775g, Tamadkala, Iran), saccharin sodium (trace sweetener; <155 mg, Tamadkala, Iran), and *thyme* hydrosol (4.65mL, Golabe-Rayehe, Iran) in distilled water; it should be emphasized that neither carboxymethyl cellulose nor saccharin sodium is topically absorbed or has therapeutic activity. The small amount of *thyme* hydrosol in the placebo serves only to replicate the aroma of the active Pedicough mouthwash.

### Sample size calculation

The sample size for this randomized controlled trial was determined to achieve 80% power at a 5% significance level based on an anticipated mean difference of 2.281 and a standard deviation of 2.563. This calculation indicated a minimum of 12 participants per group. To account for a potential 20% dropout rate, the sample size was increased to 15 participants per group, resulting in a total recruitment of 45 subjects.

### Randomization and Blinding

Participants were randomly allocated into three groups using a computer-generated random number table. Codes were securely stored in sealed, sequentially numbered envelopes, which were opened only after SRP. The study used a double-blind design; a ward nurse managed group allocation and randomization. The clinician conducting scaling and measurements remained blind to group assignments. Mouthwashes were provided in identical packaging to ensure the blinding of the participants, administrators, and clinicians.

### Treatment Protocol

The phase I of periodontal therapy, including oral hygiene instruction and thorough SRP using ultrasonic and hand instruments, followed by polishing, was performed for all the participants by a single clinician. Reducing plaque levels to zero through scaling and polishing was crucial before using the mouthwash. This step ensures uniform conditions for all patients and enhances the mouthwash's effectiveness, as it prevents new plaque formation but cannot remove the existing plaque. This approach is a standard recommendation in clinical practice [ 21 , 26 - 27 ]. 

The participants were then randomly divided into three groups (15 patients in each group) to use one of the three types of mouthwash as Group 1: CHX mouthwash (chlorhexidine 0.2%, Iran Najo, Iran), Group 2: Pedicough mouthwash, and Group 3: Placebo. Participants used 15 mL of the prescribed mouthwash twice daily for 30 seconds and 2 weeks. They were instructed to refrain from eating, drinking, or brushing for at least one hour after use. At baseline (before SRP) and after 2 weeks of mouthwash consumption, clinical periodontal parameters were measured by a single operator, blind to the group allocation. The parameters assessed included the gingival index (GI) as described by Loe and Silness, the bleeding index (BI) according to Lenox, and the plaque indices (PI) as defined by Quigley- Hein and O’Leary, evaluated across the full mouth [ 28 ]. Statistical analysis for intragroup comparisons of the PI, GI and BI from baseline to the end of the study was conducted using paired sample t-tests. Inter-group comparisons of the above parameters were analyzed using SPSS software (version 21), the Wilcoxon Signed-Rank test, and Kruskal-Wallis test. *p* Values less than 0.05 were considered statistically significant. 

## Results

**Figure 1:**
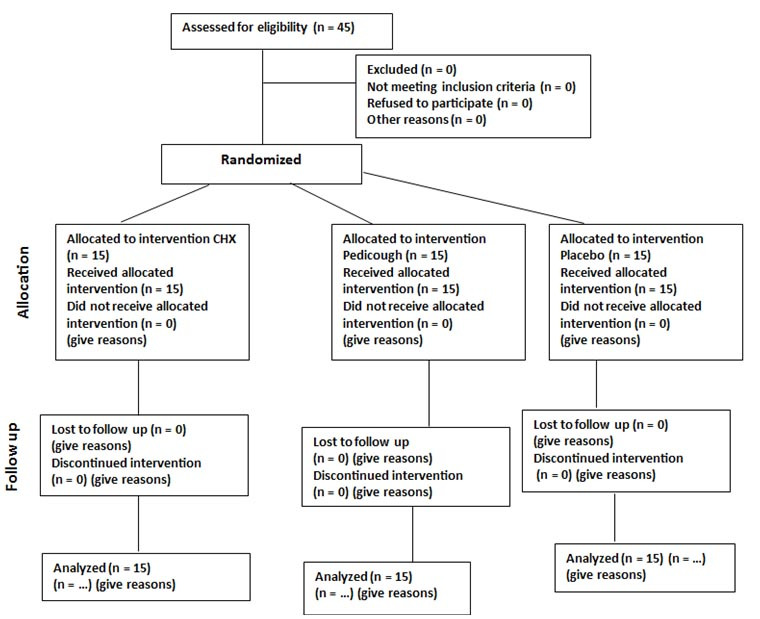
Flow diagram of study participants in each phase. (This figure illustrates the flow of participants through each phase of the study, including recruitment, allocation to intervention groups (Chlorhexidine (CHX), Pedicough, and Placebo), follow-up, and analysis. The diagram adheres to CONSORT guidelines and provides a visual representation of participant inclusion and exclusion)

A total of 45 patients (19 males and 26 females) participated in this study, which was conducted from February to March 2022 in the Department of Periodontology at Shiraz Dental School. The mean age of the study group was 31.6 years (age range: 28 to 36 years). All 45 participants completed the study protocol. They were evenly allocated into three groups according to the mouthwash used: 15 participants received CHX mouthwash, 15 received Pedicough mouthwash, and 15 received a placebo mouthwash (Figure 1). 

**Figure 2:**
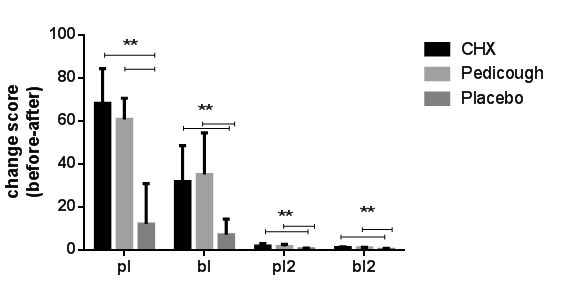
Intergroup comparison of all indices from baseline to the end of the study. (This figure shows intergroup comparisons of the Gingival Index (GI), Bleeding Index (BI), and Plaque Indices (PI) from baseline to two weeks post-treatment. Data are displayed as mean ± standard deviation (SD) for each group (Chlorhexidine (CHX), Pedicough, and Placebo). Significant changes over time and between groups are highlighted)

**Figure 3:**
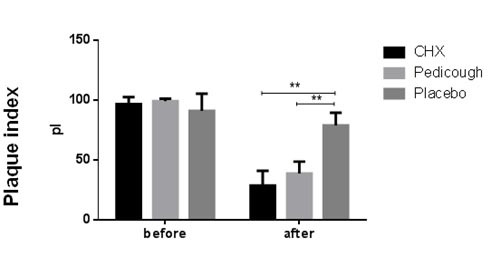
Chlorhexidine (CHX), Pedicough, and Placebo. Data are presented as mean ± standard deviation (SD), and significant differences are indicated)

**Figure 4:**
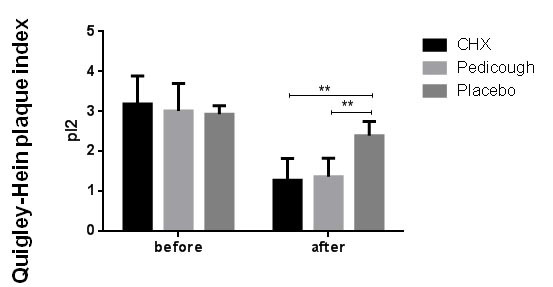
Comparison of plaque index (Quigley-Hein) at baseline and two weeks after treatment between the three Groups. (This figure shows the comparison of the Plaque Index (PI), measured using the Quigley-Hein method, at baseline and two weeks post-treatment for the Chlorhexidine (CHX), Pedicough, and Placebo groups. Results are displayed as mean ± standard deviation (SD), with statistical significance highlighted where applicable)

**Figure 5:**
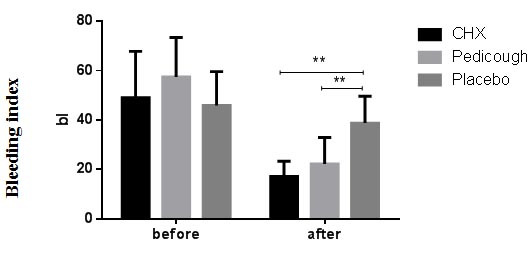
Chlorhexidine (CHX), Pedicough, and Placebo. Data are expressed as mean±standard deviation (SD), with intergroup and intragroup differences highlighted for statistical significance)

**Figure 6:**
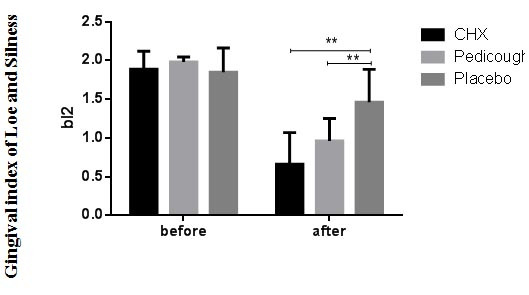
Chlorhexidine (CHX), Pedicough, and Placebo. Data are presented as mean ± standard deviation (SD). Statistical analysis highlights significant differences within and between groups, with a *p* value < 0.05 considered significant)

The comparison of clinical parameters at baseline among the three groups showed no significant differences (Table 1). Intergroup comparisons of indices from baseline to the end of the study are shown in Figure 2. The mean PI decreased in all groups from baseline to the end of the study (Table 2, Figure 3). Intergroup comparisons showed a significant difference between the placebo group and both the CHX and Pedicough groups (*p*< 0.001). However, no significant difference was found between the CHX and Pedicough groups (*p* = 0.276). The mean PI (Quigley-Hein) decreased in all groups from baseline to the end of the study (Table 3, Figure 4). Intergroup comparisons showed significant differences between the placebo group and both the CHX and Pedicough groups (*p*= 0.000). However, no significant difference was observed between the CHX and Pedicough groups (*p*= 0.100). The mean BI decreased in all groups from baseline to the end of the study (Table 4, Figure 5). Intergroup comparisons revealed a significant difference between the placebo group and both the CHX and Pedicough groups (*p*=0.000 and *p*= 0.040, respectively). However, there was no significant difference between the CHX and Pedicough groups (*p*= 0.536). The mean GI decreased in all groups from baseline to the end of the study (Table 5, Figure 6). Intergroup comparisons indicated significant differences between the placebo group and both the CHX and Pedicough groups (*p*= 0.000 and *p*= 0.007, respectively). However, no significant difference was observed between the CHX and Pedicough groups (*p*= 0.745). 

**Table 1:** Comparison of clinical parameters between groups at Vaseline

	Groups			*p* Value
	G1 Median (First 25%-third 25%)	G2 Median (First 25%-third 25%)	G3 Median (First 25%-third 25%)	
Plaque index O’Leary	100(94-100)	100(100-100)	100(80-100)	.163
Bleeding index	56(30-63)	57(54-67)	44(40-57)	.125
Plaque index Quigley Hein	3.00(2.80-3.60)	2.75(2.50-3.60)	2.83(2.80-3.00)	.236
Gingival index	2.00(1.90-2.00)	2.00(2.00-2.00)	2.00(1.83-2.00)	.209

The table compares the baseline values of clinical parameters, including Gingival Index (GI), Bleeding Index (BI), and Plaque Index (PI), across the three groups (CHX, Pedicough, and Placebo). Data are presented as mean ± standard deviation (SD). Statistical analysis was performed using ANOVA, and *p* Values < 0.05 indicate statistically significant differences

**Table 2:** Comparison of plaque index (O’Leary) two weeks after treatment between the three groups

	Groups			*p* Value		
	G1 Median (Q1-Q3)	G2 Median (First 25%-third 25%)	G3 Median (First 25%-third 25%)	1-2	1-3	2-3
Plaque index O’Leary	26.00 (23.00-35.00)	35.00 (30.00-45.00)	69 (72.00-88.00)	0.276	0.000	0.000

This table presents the comparison of Plaque Index (PI) values two weeks after treatment across the three groups: Chlorhexidine (CHX), Pedicough, and Placebo. Data are expressed as mean ± standard deviation (SD). Statistical analysis was performed using ANOVA and Tukey post hoc tests to determine intergroup differences. A *p* Value < 0.05 was considered statistically significant

**Table 3:** Comparison of plaque index (Quigley-Hein) two weeks after treatment between the three groups

	Groups			*p* Value		
	G1 Median (First 25%-third 25%)	G2 Median (First 25%-third 25%)	G3 Median (First 25%-third 25%)	1-2	1-3	2-3
Quigley-Hein plaque index	1.500(.800-1.660)	1.330(1.160-1.660)	2.330(2.160-2.660)	0.100	0.000	0.000

This table compares Plaque Index (PI) values measured using the Quigley-Hein method two weeks after treatment among the three groups: Chlorhexidine (CHX), Pedicough, and Placebo. Data are expressed as mean ± standard deviation (SD). Statistical analysis was conducted using ANOVA and Tukey post hoc tests. A *p*-value < 0.05 was considered statistically significant

**Table 4:** Comparison of Bleeding Index (BI) Two Weeks After Treatment Between the Three Groups

	Groups			*p* Value		
	G1 Median (First 25%-third 25%)	G2 Median (First 25%-third 25%)	G3 Median (First 25%-third 25%)	1-2	1-3	2-3
Bleeding index	16.00(14.00-22.00)	22.00(17.00-26.00)	40.00(32.00-48.00)	0.536	0.000	0.040

This table presents the comparison of Bleeding Index (BI) values two weeks after treatment across the three groups: Chlorhexidine (CHX), Pedicough, and Placebo. Data are reported as mean ± standard deviation (SD). Statistical differences were evaluated using ANOVA and Tukey post hoc tests. A *p* Value < 0.05 indicates statistically significant differences

**Table 5:** Comparison of Gingival Index (GI) Two Weeks After Treatment Between the Three Groups

	Groups			*p* Value		
	G1 Median (First 25%-third 25%)	G2 Median (First 25%-third 25%)	G3 Median (First 25%-third 25%)	1-2	1-3	2-3
Gingival index of Loe and Silness	.800(.330-1.00)	.83(.80-1.16)	1.25(1.16-1.83)	0.745	0.000	0.007

This table provides a comparison of Gingival Index (GI) values two weeks after treatment among the three groups: Chlorhexidine (CHX), Pedicough, and Placebo. Data are shown as mean ± standard deviation (SD). Statistical analysis included ANOVA and Tukey post hoc tests, with a *p* Value < 0.05 considered significant

## Discussion

Plaque is the primary etiological factor in periodontal disease, necessitating its regular removal to maintain healthy periodontal tissues. While mechanical plaque control, such as brushing and flossing, remains the gold standard for oral hygiene procedures, chemical agents are widely used as adjuncts to enhance the effectiveness of plaque removal. These agents are typically delivered via mouthwashes or toothpastes [ 29 ]. 

CHX, developed in 1950, remains one of the most effective antiplaque agents in dentistry. CHX is regarded as the gold standard due to its potent antibacterial properties [ 30 ]. However, the side effects that are associated with its long-term use, including staining of teeth, altered taste, calculus buildup, and potential mucosal irritation, highlight the need for alternative formulations with comparable or superior efficacy and fewer side effects [ 31 - 32 ].

Herbal products derived from medicinal plants have attracted significant attention in dental care due to their natural bioactive compounds, which exhibit anti-inflammatory, antimicrobial, and antioxidant properties. These qualities make them promising candidates for preventive and therapeutic applications in reducing plaque and managing gingivitis [ 6 ]. A review by Tidke *et al*. [ 20 ] suggests that herbal mouthwashes can be as effective as non-herbal alternatives in reducing dental plaque over the short term periods, though the evidence comes from studies of limited quality. Similarly, an *in vitro* and *ex vivo* study by Pathan *et al*. [ 5 ] compared the antimicrobial activity of the herbal mouthwash HiOra with CHX mouthwash against periodontal pathogens. While CHX demonstrated greater antimicrobial efficacy, HiOra also proved effective against the tested bacterial species under both *in vitro* and *ex vivo* conditions. Furthermore, a clinical study by Prasad *et al*. [ 12 ] assessed the anti-plaque efficacy of HiOra in comparison to CHX. The study reported comparable anti-plaque effectiveness for both mouthwashes, with HiOra presenting the additional advantage of no reported side effects. These findings highlight a growing shift towards considering herbal mouthwashes as practical alternatives to chemical formulations. This study focused on evaluating a novel herbal mouthwash, derived from Pedicough syrup containing extracts from *T. vulgaris*, *A. officinalis*, and *H. helix*. The se-lection of these plants was based on their known medicinal properties, such as antibacterial, anti-inflammatory, and antifungal activities, demonstrated primarily through *in vitro* and animal studies [ 18 , 24 - 25 ]. A 55% dilution was chosen as the starting concentration, based on prior screening studies showing that diluted thymol-based mouthwashes remain effective at lower concentrations. Patients were instructed to gargle and retain the mouthwash for 30 seconds. This initial concentration was also in-tended to minimize the risk of potential allergic reactions [ 33 - 35 ].

*T. vulgaris* and *H. helix* are medicinal plants with significant respiratory benefits. The essential oil of *T. vulgaris*, particularly its phenolic components thymol and carvacrol, exhibits potent antiseptic and bactericidal properties, aiding clearance of respiratory infections and mucus. The ethanolic leaf extract of *H. helix* contains bioactive saponins, α-hederin and hederacoside C, which underpin its therapeutic effects. The α-Hederin exerts bronchospasmolytic effects through β₂-adrenergic receptor interaction and enhances mucus clearance via the gastropulmonary reflex. Hederacoside C suppresses pro-inflammatory cytokines, delivering robust anti-inflammatory activity. Both saponins demonstrate antimicrobial efficacy against *Staphylococcus aureus*-induced infections. Critically, thymol synergistically enhances the anti-inflammatory and antimicrobial properties of *H. helix*'s saponins. These mechanisms highlight *H. helix* as a promising natural intervention for respiratory and inflammatory pathologies [ 36 - 37 ]. 

*A. officinalis*, which is historically used for respiratory diseases, is effective in treating dry coughs and when paired with other plants; helps manage various types of cough. Its active compound, rhamnogalacturonan, suppresses coughing similarly to codeine but is less effective in inflammatory conditions and does not impact airway smooth muscle or exhibit bronchodilatory effects [ 38 ]. A.officinalis also contributes to the observed anti-gingivitis effect through its mucilage and polysaccharide content, which stimulate epithelial cell regeneration on damaged and inflamed tissues, thereby promoting wound healing [ 39 ]. Additionally, the antibacterial and antifungal properties of *A. officinalis* are attributed to its flavonoid and phenolic acid constituents [ 40 - 41 ]. 

*T. vulgaris*, *A. officinalis*, and *H. helix* were chosen for their individual therapeutic potential, each supported by a body of scientific evidence. *T. vulgaris* is known for its antimicrobial properties, attributed to active compounds such as thymol and carvacrol, as well as its ability to modulate inflammatory pathways. Studies have demonstrated its antioxidant effects and its role in reducing inflammatory responses in both *in vitro* and animal models [ 36 , 42 ] The study by Fani *et al*. [ 24 ] demonstrated the potent antimicrobial activity of *T. vulgaris* oil against clinical isolates of *Streptococcus pyogenes, Streptococcus mutans, Candida albicans, Aggregatibacter actinomycetemcomitans,* and *Porphyromonas gingivalis* *in vitro*. These findings suggest its potential application in mouth rinses, toothpastes, and aromatherapy products for preventing and treating oral infections caused by these pathogens. Similarly, *H. helix* has shown potent anti-inflammatory and analgesic effects in acute and chronic inflammation models, alongside its antibacterial properties [ 16 , 25 , 43 ]. Lastly, *A. officinalis* is renowned for its mucosal-protective effects and has demonstrated broad-spectrum antimicrobial activity against periodontal pathogens. Furthermore, it has analgesic and anti-inflammatory effects [ 19 , 44 - 45 ]. 

This study demonstrated significant improvements in plaque control, gingival bleeding, and inflammation in both the herbal mouthwash and CHX groups compared to the placebo group. While all groups, including the placebo group, showed some degree of improvement, the superior performance of the test groups suggests that the herbal formulation could be an effective alternative to CHX. The placebo group’s improvement can be attributed to the benefits of SRP and the positive behavioral impact of oral hygiene instructions. Additionally, while participants modify their behavior due to awareness of being observed, the Hawthorne effect may have contributed to the progress of the placebo group [ 46 ]. 

Studies that used one or more ingredients of Pedicough have shown promising results [ 47 - 49 ]. Radvar *et al*. [ 49 ] demonstrated that an herbal mouthwash containing *Salix alba, Malva sylvestris*, and *A. officinalis* offered clinical benefits comparable to CHX when used as an adjunct to SRP, particularly in patients with gingivitis. Kręgielczaka *et al*. [ 48 ] showed that herbal mouth rinses combining flaxseed (*Linum usitatissimum*), chamomile (*Matricariae flos*) and marshmallow (*A. officinalis*) offer effective anti-inflammatory, antioxidant, and protective coating properties, making them a cost-effective and accessible option for managing oral mucosal conditions such as xerostomia, oral lichen planus, and burning mouth syndrome. These rinses alleviate dryness, burning, and pain while promoting moisture and healing, providing a valuable supplement to daily oral hygiene and treatment practices.

Ghorbani *et al*. [ 47 ] demonstrated that a mouthwash containing *A. officinalis* root extract had greater effectiveness in managing chemotherapy-induced stomatitis compared to standard mouthwash solutions. This suggests that *A. officinalis* could be a valuable adjunct therapy to reduce the incidence of stomatitis when used alongside chemotherapy agents. Pezeshkian *et al*. [ 50 ] demonstrated that an herbal mouthwash containing *Cyperus rotundus* and *T. vulgaris* extracts exhibited significant antimicrobial activity against periodontal pathogens, outperforming CHX in preventing biofilm formation and showing comparable biofilm destruction. This suggests its potential as an effective alternative with fewer side effects.

Despite the promising individual properties of these plants, this study is among the first to evaluate their combined efficacy in dental applications. The findings suggest that the combined effects of *T. vulgaris*, *H. helix*, and *A. officinalis* can significantly reduce plaque and gingival inflammation when compared to placebo, potentially matching or even exceeding the positive performance of CHX without the adverse effects commonly associated with CHX use. However, further studies are needed to isolate and evaluate the individual contributeons and any synergistic interactions of these components [ 31 ]. Notably, during the two-week study, the herbal mouthwash group experienced no adverse effects, such as tooth staining, taste changes, or mucosal irritation. 

The current study also highlights limitations in the use of mouthwashes for plaque control. Oral biofilms, the primary structure of plaque, form a protective barrier that reduces the effectiveness of chemical agents. This inherent limitation means that mouthwashes should not be used in isolation but as adjuncts to mechanical cleaning methods like brushing and flossing [ 51 ]. For individuals with periodontal pockets or advanced gingival inflammation, direct subgingival delivery methods, such as irrigation or drug-release devices, may be necessary for effective microbial control [ 52 ]. 

Additionally, the study’s two-week duration, while sufficient to observe short-term effects, limits the ability to assess the long-term efficacy of the herbal mouthwash. According to the ADA, clinical trials evaluating gingivitis control should ideally span six months [ 53 ]. Short time studies, such as the current one, may miss potential side effects or longer-term benefits. However, the shortened evaluation period in this study was intentional, aimed at avoiding the known side effects of prolonged CHX use and addressing concerns about patient compliance over extended durations.

While CHX remains a widely accepted standard in dental care, its long-term limitations underscore the need for safer, cost-effective alternatives [ 30 ]. Herbal mouthwashes like the one tested in this study show potential to fill this gap, offering comparable benefits without the associated drawbacks. However, further research is needed to validate these findings on a larger scale and over longer durations. Future studies should also explore the optimization of herbal formulations and investigate additional plant-based agents with antimicrobial and anti-inflammatory properties.

### Limitations and suggestions

This study, which explored the use of an oral syrup as a mouthwash, serves as a foundation for future research in periodontal and oral medicine. Future studies could evaluate other herbal-based mouthwash alongside the gold standard CHX, with a focus on purifying and assessing the synergistic activity of individual components. Additionally, antimicrobial assessments and biochemical analysis of gingival crevicular fluid and saliva could be included. One limitation of this study was its short duration, primarily due to the concerns about participant compliance and the avoidance of CHX side effects. Longer trials would provide more realistic results and allow for the assessment of clinical parameters, such as pocket depth and attachment gain, which require more time to show significant changes.

## Conclusion

Despite the limitations of this study, the novel herbal mouthwash derived from Pedicough demonstrated clinical benefits similar to those of CHX when used alongside SRP in patients with gingivitis. The formulation, consisting of three plants (*T. vulgaris*, *H. helix* and *A. officinalis*), significantly improved key periodontal parameters when compared to placebo, including plaque, gingival, and bleeding indices, without any reported adverse effects. However, larger-scale studies with extended durations are necessary to confirm these findings and further evaluate the long-term efficacy and safety of this herbal mouthwash.
